# The efficacy of memory load on speech-based detection of Alzheimer’s disease

**DOI:** 10.3389/fnagi.2023.1186786

**Published:** 2023-06-02

**Authors:** Minju Bae, Myo-Gyeong Seo, Hyunwoong Ko, Hyunsun Ham, Keun You Kim, Jun-Young Lee

**Affiliations:** ^1^Interdisciplinary Program in Cognitive Science, Seoul National University, Seoul, Republic of Korea; ^2^Department of Psychiatry, SMG-SNU Boramae Medical Center, Seoul National University College of Medicine, Seoul, Republic of Korea; ^3^Samsung Medical Center, Samsung Advanced Institute for Health Sciences and Technology, Sungkyunkwan University, Seoul, Republic of Korea

**Keywords:** Alzheimer’s disease, Mini Mental State Examination, speech acoustics, automatic speech analysis, machine learning

## Abstract

**Introduction:**

The study aims to test whether an increase in memory load could improve the efficacy in detection of Alzheimer’s disease and prediction of the Mini-Mental State Examination (MMSE) score.

**Methods:**

Speech from 45 mild-to-moderate Alzheimer’s disease patients and 44 healthy older adults were collected using three speech tasks with varying memory loads. We investigated and compared speech characteristics of Alzheimer’s disease across speech tasks to examine the effect of memory load on speech characteristics. Finally, we built Alzheimer’s disease classification models and MMSE prediction models to assess the diagnostic value of speech tasks.

**Results:**

The speech characteristics of Alzheimer’s disease in pitch, loudness, and speech rate were observed and the high-memory-load task intensified such characteristics. The high-memory-load task outperformed in AD classification with an accuracy of 81.4% and MMSE prediction with a mean absolute error of 4.62.

**Discussion:**

The high-memory-load recall task is an effective method for speech-based Alzheimer’s disease detection.

## 1. Introduction

In the early stages of Alzheimer’s disease (AD), impairments in semantic memory and the executive component of verbal fluency manifest as language deficiencies characterized by difficulties in initiating speech and slower response times (Appell et al., 1982; Murdoch et al., 1987; Salmon and Chan, 1994; Emery, 2000; Minati et al., 2009; de Looze et al., 2022). As the disease progresses, syntactic and pragmatic language abilities begin to show impairment with speech becoming verbose, circuitous, incoherent, and exhibiting repetition of syllables, words, or sentences (Appell et al., 1982; Kempler et al., 1987; Liampas et al., 2022; Roelofs, 2023). In addition to linguistic deficiencies, research has also identified impairments in speech motor function and changes in the acoustic characteristics of speech in AD, including alterations in voice pitch, prosody, intensity, vocal quality, and speech rate (Hoffmann et al., 2009; Martínez-Sánchez et al., 2012; Sajjadi et al., 2012; Meilán et al., 2014; Cho et al., 2022).

Research on speech-based AD detection has used observable acoustic features in AD patients. For example, López-de-Ipiña analyzed speech recordings from a multilingual database, AZTIAHO, comprised of video recordings of conversation, and built AD classification models with 86.1% accuracy (López-de-Ipiña et al., 2013). Meilán characterized AD patients with 84.8% accuracy using an oral sentence reading task (Meilán et al., 2014). König used speech recordings from multiple speech tasks such as countdown, picture description, sentence repetition, and semantic fluency, to build classification models, achieving 87% accuracy in distinguishing between AD patients and healthy older adults (König et al., 2015). However, previous research has focused less on how speech tasks may effectively reflect the distinctive speech characteristics of AD and more on utilizing accessible speech data from random speech tasks.

It is important to understand the inherent properties of speech tasks, as different speech tasks demand distinct cognitive abilities that may be susceptible to AD pathology or remain intact until the final phase of AD. Recent empirical data and theoretical foundations imply that cognitive loads traditionally thought to be unrelated to speech motor performance may, in fact, have an effect on speech motor performance, as reflected in changes in speech kinematics and acoustic characteristics (Caruso et al., 1994; Dromey and Bates, 2005; Dromey and Shim, 2008; Kemper et al., 2010; Huttunen et al., 2011; Sadagopan and Smith, 2013; Bailey and Dromey, 2015; MacPherson et al., 2017). For example, the cognitive load in the form of divided attention affects speech kinematics, such as lower lip movement pattern variability, articulatory displacement and velocity, and utterance duration (Sadagopan and Smith, 2013; Bailey and Dromey, 2015). Other studies focused on acoustic findings have revealed that the increased cognitive demand is correlated with a decrease in speech rate (Kemper et al., 2010), increased cepstral peak prominence, decreased low-to-high spectral energy ratio (MacPherson et al., 2017), sound pressure level, fundamental frequency, intensity, and the variability of fundamental frequency and intensity (Dromey and Bates, 2005; Dromey and Shim, 2008; Huttunen et al., 2011). Moreover, special populations such as children, older adults, and patients with cognitive impairment, who are assumed to have comparatively smaller cognitive reserves, may be more susceptible to cognitive load-induced changes in speech motor performance (Kamhi et al., 1984; Maner et al., 2000; Prelock and Panagos, 2009; Svindt et al., 2019). Previous investigations have revealed the susceptibility of older adults to increases in cognitive load, exhibiting greater performance costs, such as reductions in fine motor control, disruptions in the stability and timing of speech, and increased articulatory coordination variability (Ramig and Ringel, 1983; Kemper et al., 2010, 2011; Marzullo et al., 2010; Bailey and Dromey, 2015; MacPherson, 2019).

Based on prior research, it is apparent that there are clear associations between cognitive load and speech motor performance. Since AD is characterized by memory impairment, a speech task requiring a high memory load would cause greater changes in the acoustic features of AD patients. Furthermore, it is expected that a speech task with a high memory load will have higher diagnostic value as a speech-based AD detection method, as well as demonstrate superior performance in AD classification and in the MMSE prediction model. In this study, we hypothesized that (1) there are differences in speech characteristics between AD patients and healthy older adults, (2) a high-memory-load speech task makes AD speech characteristics detectable, and (3) a high-memory-load speech task enhances the performance of AD classification and MMSE prediction models.

## 2. Methods

### 2.1. Participants

We collected speech data from 45 individuals with mild-to-moderate AD and 44 healthy older adults. This study was conducted in accordance with the current Declaration of Helsinki (World Medical Association, 2013). The institutional review board approved the protocol of the SMG-SNU Boramae Medical Center (IRB No. 30–2020-174) and the informed consent was exempted but we obtained the verbal consent. AD patients were recruited from the SMG-SNU Boramae Medical Center. AD was diagnosed by a geriatric psychiatrist using the National Institute of Aging and Alzheimer’s Association (NIA-AA) criteria (Hyman et al., 2012), and subjects suspected or diagnosed with dementia types other than AD were excluded. Healthy older adults were recruited from the local community. They were also screened by a geriatric psychiatrist, and the Mini-Mental State Examination (MMSE) score was 27 or higher for all participants in that group (Cockrell and Folstein, 1988). The MMSE was administered on a different day from the experiment. All participants were (1) between 65 and 85 years of age, (2) native Korean speakers, (3) had no neurological/psychiatric disorder other than AD, (4) had no hearing problem, and (5) volunteered to participate.

The demographics of participants are presented in Table 1. The final data were comprised of 45 individuals with mild-to-moderate AD (number of female participants = 28, mean age = 75.67 ± 4.88, mean education years = 7.24 ± 4.88, mean MMSE score = 16.89 ± 4.27) and 44 healthy older adults (number of female participants = 24, mean age = 75.30 ± 0.80, mean education years = 7.67 ± 5.39, mean MMSE score = 28.95 ± 1.15). There were no significant differences between sex ratio, mean age, and mean education years between groups.

**Table 1:** Demographics of participants in the dataset.

	AD (*n* = 45)	HC (*n* = 44)	Value of *p*
Gender, female (%)	28 (62.2)	24 (54.5)	0.463
Mean age, y (SD)	75.67 (4.88)	75.30 (0.80)	0.624
Mean education, y (SD)	7.24 (5.36)	7.67 (5.39)	0.708
Mean MMSE score (SD)	16.89 (4.27)	28.95 (1.15)	< 0.001

AD, Alzheimer’s disease; HC, healthy older adults; MMSE, mini-mental statement examination; value of *p*, chi-squared test was used for gender and the independent *t*-test was used for age, education, and MMSE score.

### 2.2. Speech task

Speech tasks were performed over the phone by four well-trained researchers (three researchers with master’s degree in Clinical Psychology and one graduate student in Cognitive Science). Participants were instructed to perform the tasks alone in a silent room so that they would not be interrupted. One call was conducted per each participant. It took about 10 min to conduct three speech tasks. The participants’ responses were recorded as *.m4a sound files and then converted to *.wav sound files.

In this study, three speech tasks with varying memory loads were administered: the interview task, the repetition task, and the recall task. We manipulated the memory load by varying the length of stimuli to be remembered. In the low memory load condition, participants were given the interview task, which consisted of five questions about personal information and daily life activities, to which they were free to respond. In the moderate memory load condition, the repetition task was administered. As the researcher read a sentence aloud to the participants, they were instructed to remember and repeat the sentence as precisely as they could. If a participant was unable to repeat the sentence correctly, the researcher read it aloud again. Stimuli were composed of two sets, and each set included eight sentences. In the high-memory-load condition, the recall task was performed. In this circumstance, the length of items to be remembered increases from one sentence to one paragraph. As a paragraph of the story was presented, participants were asked to recall as many details as possible. The key details of story that are expected to be answered such as the name of the main characters and the main event of the story were defined under the agreement of the researchers before the experiment. If the response was insufficient or lacked key details, the researcher encouraged the participants to elaborate up to two times (e.g., Who else are the characters in the story?, What happened to the main character?). Two stories were given, and each story was composed of eight sentences. One of them was adapted from the Kim’s study (Kim and Sung, 2014).

### 2.3. Data preparation

**Figure 1:**
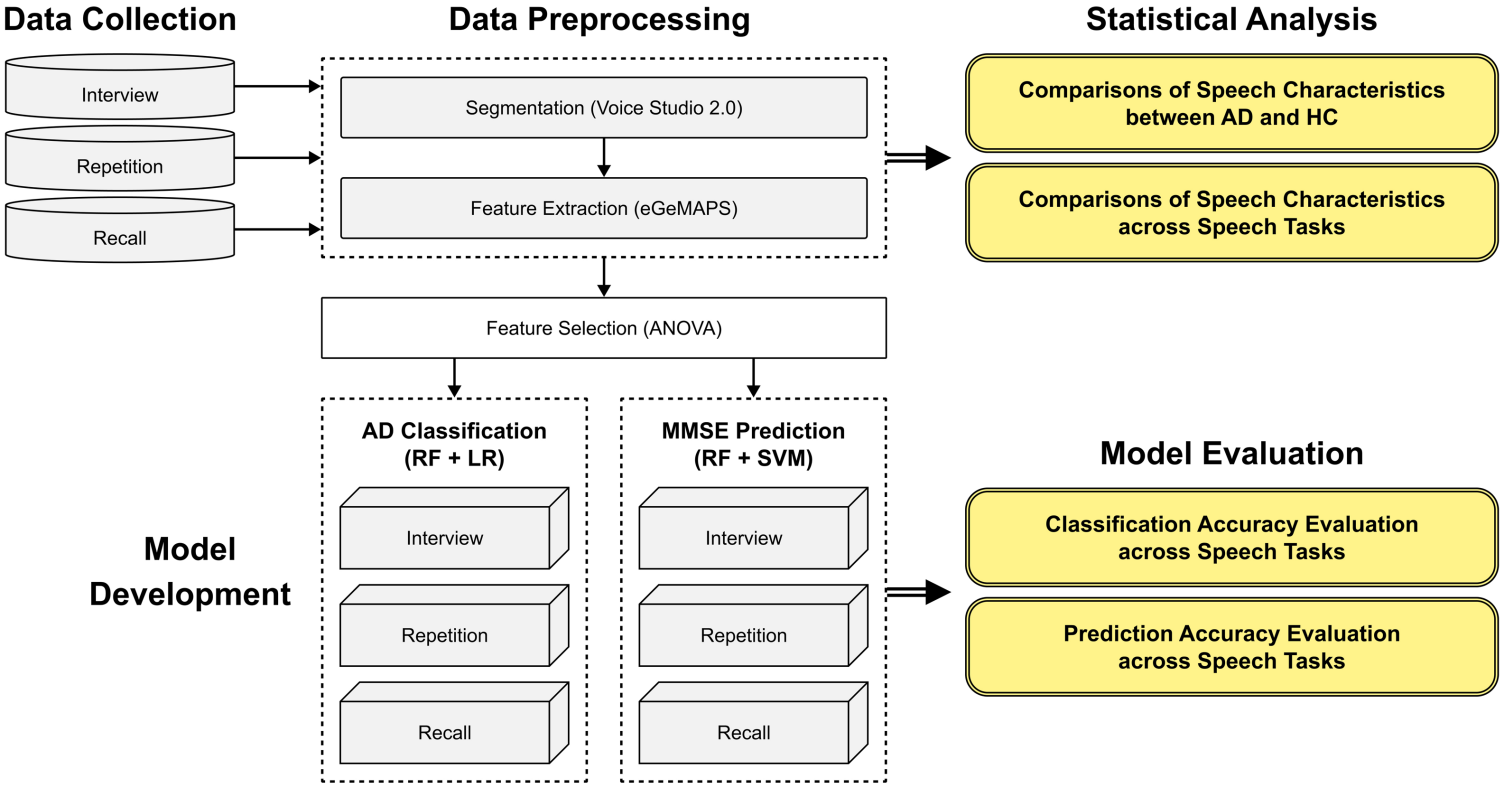
An experimental scheme of the study.

An experimental scheme of the total study is presented in Figure 1. Rather than the automatic speaker diarization, manual speech segmentation method was employed to eliminate the voice of researchers completely to overcome the speech overlap issue between researchers and participants. Using the open-source software “Voice studio2.0,” the acquired speech samples were segmented into pauses and utterances. Twenty researchers with training in audio segmentation guidelines manually tagged the time stamps of corresponding utterances, while four other researchers evaluated the quality of the time stamp tagging to ensure if the speech of participants was completely included and the speech of researchers was completely removed. Among the 3,831 speech segments, 30 samples were disqualified due to excessive noise or sound distortion.

We extracted the extended Geneva acoustic feature set (eGeMAPS; Eyben et al., 2015) using the Python library and the openSMILE toolkit (Eyben et al., 2010). The eGeMAPS is a minimalistic set of voice parameters that was initially developed to recognize the affective state of a speaker based on the information conveyed by the voice. However, eGeMAPS is now widely used in various areas of speech analysis, particularly in speech-based AD detection (Haider et al., 2019; Pappagari et al., 2021; Valsaraj et al., 2021). The eGeMAPS is utilized in numerous studies because it consists of a standardized, limited set of features that were selected based on their theoretical relevance and ability to analyze an important aspect of speech (Xue et al., 2019). Because eGeMAPS is divided into four parameter groups, namely, frequency-related, intensity-related, temporal, and spectral, it is capable of revealing significant aspects of voice characteristics. Frequency-related features are related to the pitch of speech such as Fundamental Frequency (F0), jitter. Intensity-related features are involved to the amplitude of speech such as loudness, and shimmer. Temporal features are related to the speed of speech such as the duration of speech. Spectral features indicate how much energy is present in the signal at different frequencies such as MFCC, Alpha Ratio, and Hammarberg Index.

### 2.4. Statistical analysis

We described the demographics of the participants using the independent t test or chi-squared test. We performed an independent two-sample t test or Welch’s two-sample t test to investigate differences in speech characteristics between AD patients and healthy older adults. We conducted a pairwise t test to examine the impact of memory load on the speech characteristics of AD. A two-sided *p* value of <0.05 was determined to be statistically significant, and a Bonferroni correction for multiple comparisons was applied. All statistical analyses were performed using R version 4.2.1.

### 2.5. Machine learning

In this study, feature selection was accomplished via analysis of variance (ANOVA) only on the training data. Statistically significant features (*p* < 0.01) were selected from the original features that were extracted from speech samples to build classification models. Certain features were scaled so that features with large values could not outweigh features with small values. Each dataset from the three speech tasks was split into 70% training data and 30% test data. Due to the segmentation of each participant’s speech data into utterances, the dataset contains multiple speech segments from a single participant. When splitting datasets, we ensured that there were no participant overlaps and that each set maintained a similar class distribution. A weighted voting classifier combining random forest and logistic regression was then used to classify the data. Weights were added to the classifier based on the performance of that classifier so that a classifier that achieved better accuracy was given a higher weight. The hyperparameters were tuned on training data through 10-fold cross-validation, and the resulting model was evaluated on the testing data. To evaluate and compare the performance of different speech tasks, accuracy, precision, sensitivity, specificity, F1-score, and the area under the curve (AUC) were measured.

We built MMSE prediction models using the same methodology as the AD classification models. A weighted voting regressor combining random forest, support vector machine was built. To evaluate and compare the prediction performance of speech tasks, the mean absolute error (MAE), and the root mean square error (RMSE) were calculated. Machine learning was performed using Python version. 3.10.8.

## 3. Results

### 3.1. Speech characteristics of AD

To investigate the speech characteristics of AD, frequency, intensity, and temporal features were compared between patients with AD and healthy older adults. While the eGeMAPS includes spectral features, because they are timbral features, it is difficult to gain intuitive insight from them, even if AD patients have spectral features that differ from those of healthy older adults. This is why the present study investigated frequency, intensity, and temporal characteristics to determine the speech characteristics of AD.

The comparisons of acoustic features between AD patients (AD) and healthy older adults (HC) are summarized in Table 2. First, for the frequency-related features, the mean F0 (AD = 30.14 ± 3.13, HC = 31.6 ± 3.94, *p* < 0.001), 20th percentile of F0 (AD = 26.31 ± 3.15, HC = 28.55 ± 4.98, *p* < 0.001), and 50th percentile of F0 (AD = 30.16 ± 3.35, 31.32 ± 4.02, *p* = 0.011) indicate the pitch of the voice. In all cases, values are lower for AD patients. The F0 percentile range indicates that the F0 range for AD patients was greater (AD = 7.4 ± 1.77, HC = 5.73 ± 3.36, *p* < 0.001) and high standard deviation of F0 also indicates the higher variability of F0 in AD patients (AD = 0.19 ± 0.04, HC = 0.16 ± 0.06, *p* < 0.001). AD patients demonstrated a greater degree of jitter (AD = 0.04 ± 0.01, HC = 0.03 ± 0.02, *p* < 0.001), indicating greater pitch variability. To sum up, frequency-related features indicate that AD patients have lower voice pitch and higher pitch variability compared to healthy older adults.

**Table 2:** A comparison in acoustic features between patients with AD and healthy older adults.

Speech Features		AD	HC	*p*
Frequency	F0 percentile range	7.4 (1.77)	5.73 (3.36)	<0.001
	20th percentile of the F0	26.31 (3.15)	28.55 (4.98)	<0.001
	Jitter mean	0.04 (0.01)	0.03 (0.02)	<0.001
	Normalized F0 SD	0.19 (0.04)	0.16 (0.06)	<0.001
	F0 mean	30.14 (3.13)	31.6 (3.94)	<0.001
	50th percentile of the F0	30.16 (3.35)	31.32 (4.02)	0.011
Intensity	Loudness peaks per second	2.37 (0.57)	2.82 (0.59)	<0.001
	Loudness percentile range	0.46 (0.25)	0.62 (0.28)	<0.001
	Loudness falling slope mean	4.6 (2.25)	5.73 (2.64)	<0.001
	80th percentile of the loudness	0.55 (0.27)	0.68 (0.28)	<0.001
	Loudness rising slope mean	5.33 (2.61)	6.55 (2.82)	<0.001
	Loudenss falling slope SD	3.03 (1.41)	3.56 (1.67)	0.006
	50th percentile of the loudness	0.22 (0.14)	0.27 (0.12)	0.008
	Loudness mean	0.33 (0.15)	0.37 (0.14)	0.012
	Loudness rising slope SD	3.75 (1.69)	4.27 (1.96)	0.023
Temporal	Mean duration of voiced region	0.3 (0.11)	0.37 (0.14)	<0.001
	SD duration of voiced region	0.24 (0.08)	0.28 (0.08)	<0.001
	Number of voiced region	2.06 (0.4)	1.79 (0.82)	<0.001

AD, Alzheimer’s disease; HC, healthy older adults; *p*, independent *t*-test, or Welch’s two-sample test were used as appropriate and a Bonferroni correction for multiple comparisons was applied; Features are sorted by value of *p*.

For AD patients, the 80th percentile of loudness (AD = 0.55 ± 0.27, HC = 0.68 ± 0.28, *p* < 0.001), 50th percentile of loudness (AD = 0.22 ± 0.14, HC = 0.27 ± 0.12, *p* = 0.008) and the mean loudness (AD = 0.33 ± 0.14, HC = 0.37 ± 0.14, *p* = 0.012) were lower. Loudness peaks per second (AD = 2.37 ± 0.57, HC = 2.82 ± 0.59, *p* < 0.001), loudness percentile range (AD = 0.46 ± 0.25, HC = 0.62 ± 0.28, *p* < 0.001), loudness falling slope (AD = 4.6 ± 2.25, HC = 5.73 ± 2.64, *p* < 0.001), and loudness rising slope (AD = 5.33 ± 2.61, HC = 6.55 ± 2.82, *p* < 0.001), all of which indicating loudness variability, were reduced in AD patients. Intensity-related features indicate that AD patients exhibit diminished loudness and monotonous loudness.

In terms of temporal features, AD patients had a shorter duration of voiced regions (AD = 0.3 ± 0.11, HC = 0.37 ± 0.14, *p* < 0.001) and a greater number of voiced regions (AD = 2.06 ± 0.4, HC = 1.79 ± 0.82, *p* < 0.001). This suggests that AD patients had difficulty maintaining speech, spoke at a slower rate, and produced more pauses and hesitations than healthy older adults.

### 3.2. The effect of memory load on the speech characteristics of patients with AD

To investigate the effect of memory load on the speech characteristics of patients with AD, we divided the dataset according to the applied speech task and conducted an independent t test. Table 3 depicts the speech characteristics across speech tasks that demonstrated statistical significance in at least one of the study tasks. The greatest number of recall task features was reported to be statistically significant (*n* = 21). In the interview and recall tasks, fourteen and four features, respectively, demonstrated statistical significance.

**Table 3:** A comparison of speech characteristics between AD patients and healthy older adults across speech tasks.

Speech Features		Interview			Repetition			Recall		
		AD	HC	*p*	AD	HC	*p*	AD	HC	*p*
Frequency	Normalized jitter SD	1.54 (0.21)	1.65 (0.2)	**0.011**	1.7 (0.21)	1.74 (0.23)	0.464	1.59 (0.24)	1.77 (0.27)	**<0.001**
	F0 percentile range	8.52 (1.79)	6.53 (3.37)	**<0.001**	6.12 (1.49)	4.8 (2.99)	**0.011**	7.56 (1.62)	5.85 (3.33)	**0.003**
	Jitter mean	0.04 (0.01)	0.04 (0.01)	**0.016**	0.04 (0.01)	0.03 (0.01)	0.101	0.04 (0.01)	0.03 (0.02)	**0.007**
	20th percentile of the F0	26.48 (3.24)	29.14 (5.31)	**0.006**	26.59 (3.19)	28.12 (4.2)	0.059	25.87 (3.01)	28.41 (5.43)	**0.008**
	F0 Rising slope SD	280.29 (133.09)	314.12 (106.09)	0.190	305.72 (139.15)	286.51 (121.58)	0.492	318.75 (182.34)	401.88 (138.27)	**0.018**
	F0 Falling slope SD	129.3 (53.47)	110.27 (81.46)	0.201	126.13 (65.76)	114.29 (64.86)	0.397	133.21 (126.57)	184.22 (75.89)	**0.024**
	F0 mean	30.86 (3.11)	32.52 (4.08)	**0.035**	29.85 (3.1)	30.76 (3.25)	0.181	29.72 (2.99)	31.51 (4.38)	**0.029**
	Normalized F0 SD	0.19 (0.04)	0.16 (0.06)	**0.010**	0.17 (0.04)	0.15 (0.05)	0.105	0.19 (0.04)	0.17 (0.06)	**0.030**
	50th percentile of the F0	30.98 (3.36)	32.31 (4.08)	0.100	30.07 (3.4)	30.54 (3.08)	0.501	29.43 (3.11)	31.12 (4.67)	**0.050**
Loudness	Loudness percentile range	0.49 (0.27)	0.64 (0.28)	**0.016**	0.52 (0.24)	0.6 (0.31)	0.168	0.38 (0.25)	0.64 (0.22)	**<0.001**
	Loudness peaks per second	2.39 (0.53)	2.86 (0.56)	**<0.001**	2.68 (0.51)	2.96 (0.47)	**0.009**	2.05 (0.62)	2.64 (0.55)	**<0.001**
	80th percentile of the loudness	0.58 (0.28)	0.69 (0.28)	0.071	0.61 (0.26)	0.66 (0.31)	0.475	0.46 (0.26)	0.68 (0.21)	**<0.001**
	Loudness mean	0.35 (0.16)	0.39 (0.15)	0.195	0.36 (0.14)	0.36 (0.16)	0.936	0.28 (0.14)	0.37 (0.12)	**0.001**
	Loudness falling slope mean	4.88 (2.37)	6.01 (2.88)	**0.048**	4.45 (2.02)	5.05 (2.49)	0.221	4.46 (2.23)	6.14 (2.58)	**0.002**
	Normalized shimmer SD	0.73 (0.1)	0.8 (0.11)	**0.004**	0.78 (0.09)	0.84 (0.13)	**0.019**	0.75 (0.09)	0.83 (0.12)	**0.002**
	20th percentile of the loudness	0.09 (0.03)	0.06 (0.07)	**0.003**	0.1 (0.03)	0.06 (0.07)	**0.001**	0.08 (0.03)	0.04 (0.07)	**0.003**
	Loudness rising slope mean	5.68 (2.7)	7.03 (3.11)	**0.032**	5.13 (2.34)	5.72 (2.64)	0.272	5.16 (2.65)	6.9 (2.73)	**0.003**
	50th percentile of the loudness	0.22 (0.15)	0.27 (0.12)	0.105	0.27 (0.13)	0.28 (0.13)	0.810	0.17 (0.13)	0.25 (0.1)	**0.004**
	Loudness falling slope SD	3.24 (1.46)	3.71 (1.76)	0.171	2.77 (1.17)	3.01 (1.54)	0.414	3.09 (1.44)	3.95 (1.71)	**0.013**
Temporal	Mean duration of voiced region	0.3 (0.12)	0.38 (0.14)	**0.003**	0.32 (0.09)	0.37 (0.13)	0.059	0.29 (0.1)	0.35 (0.14)	**0.014**
	SD duration of voiced region	0.23 (0.09)	0.28 (0.08)	**0.008**	0.25 (0.05)	0.27 (0.06)	0.067	0.25 (0.08)	0.3 (0.1)	**0.011**

AD, Alzheimer’s disease; HC, healthy older adults; *p*, independent *t*-test, or Welch’s two-sample test were used as appropriate and a Bonferroni correction for multiple comparisons was applied; Features are sorted by value of *p*; Statistically significant features are in bold.

Regarding frequency-related features, we observed a certain tendency in the speech of AD patients regardless of the speech tasks. In all speech tasks, AD patients demonstrated a lower pitch (e.g., F0 mean, 20th, and 50th percentile of the F0) and greater pitch variability (F0 percentile range, Normalized F0 SD). In the interview and repetition tasks, however, those tendencies were not recognizable enough to report statistical significance; in the recall task, however, the majority of features demonstrated statistical significance. In addition, when we previously analyzed speech features regardless of speech task, there were no significant differences between groups for the F0 rising slope and F0 falling slope. However, after separating the data by speech task, it was discovered that these features were significantly different for the recall task.

In the case of intensity-related features, we also observed a consistent trend regardless of the speech tasks. In all speech tasks, patients with AD exhibited small loudness (loudness mean, 20th, 50th, and 80th percentile of the loudness) and monotonous loudness (loudness percentile range, loudness peaks per second, loudness rising slope, and loudness falling slope). Despite this, the interview and repetition task failed to identify statistically significant differences in the majority of intensity-related features. In the meantime, they were distinguishable enough for statistical significance in the recall task.

In terms of temporal features, the recall task did not show superior discriminability. AD patients exhibited a shorter duration and a higher number of breaks in the interview and the recall task.

### 3.3. Machine learning performances

**Figure 2:**
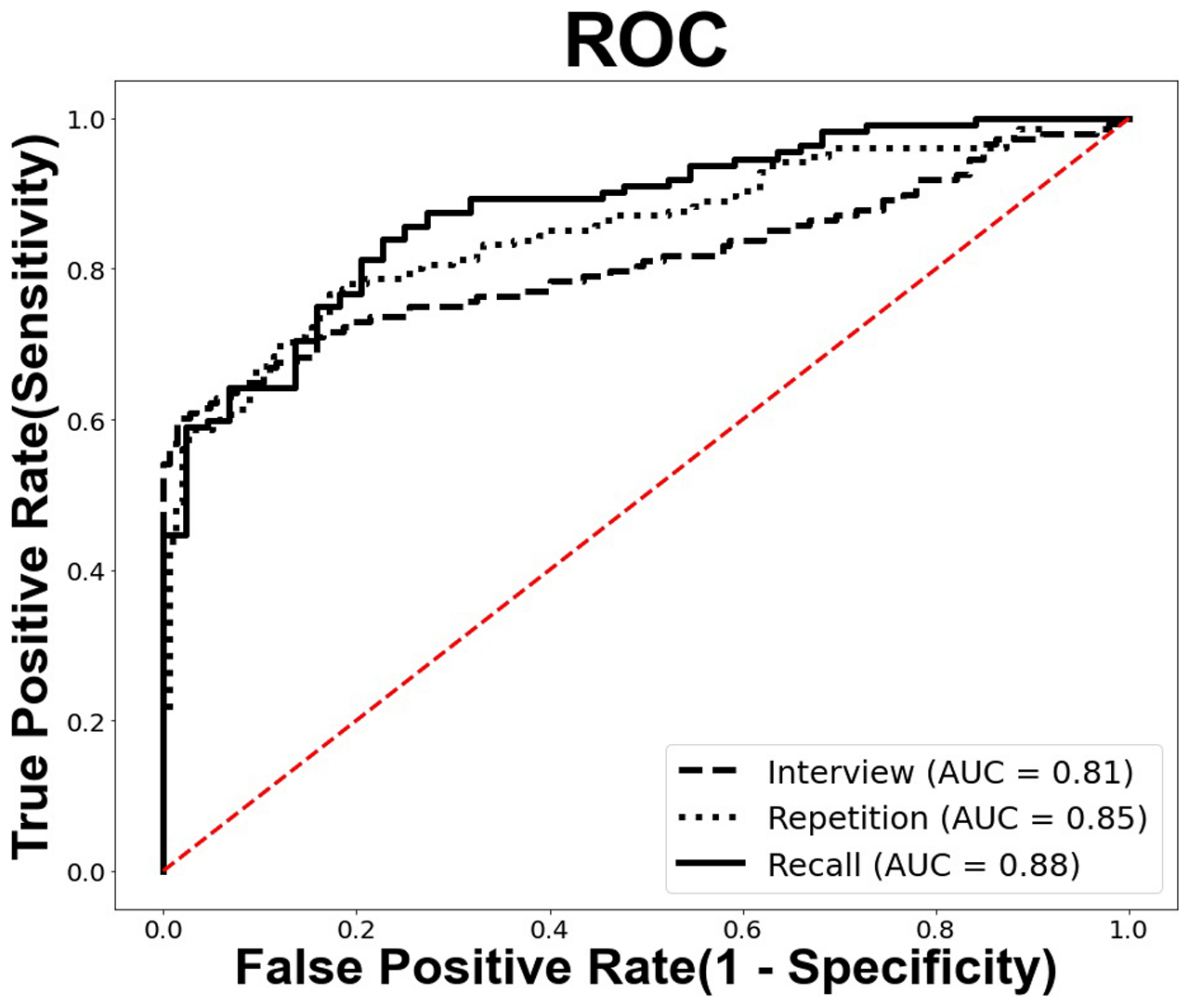
The ROC curves of the recall, repetition, and interview speech tasks. ROC, receiver operating characteristic; AUC, area under the curve.

The diagnostic value of the three speech tasks was evaluated, and the results are displayed in Table 4; Figure 2. In Table 4, the performance of the AD classification models is compared. With an accuracy of 81.4%, the recall task outperformed all other speech tasks, including interviews and repetitions. The repetition task achieved the second-highest accuracy of 78.5%, and the interview task achieved 76.1%.

**Table 4:** A comparison of the performance of AD classification models.

Task	Accuracy	Precision	Sensitivity	Specificity	F1-score
Interview	76.1	77.9	73.6	78.6	75.7
Repetition	78.5	78.6	78.1	**79.0**	78.3
Recall	**81.4**	**90.3**	**83.0**	77.3	**86.5**

The performances of AD classification of three speech tasks are presented. A weighted voting classifier combining the logistic regression and random forest was used. The best results are given in bold.

The recall task demonstrates the highest scores for all other performance analysis metrics, including precision, sensitivity, and F1-score, but specificity, indicating the relative success of AD classification. In particular, the recall task has both high F1-score of 86.5%, indicating an exceptional ability to screen AD patients correctly and not miss AD patients.

Moreover, an analysis was conducted on the Area Under the Curve (AUC) to evaluate the classification performance in distinguishing between classes (see Figure 2). The recall task yielded the highest AUC value of 0.88, while the interview and repetition tasks yielded AUC values of 0.81 and 0.85, respectively.

In the MMSE prediction, we measured MAE, and RMSE, to evaluate the model performances. The performance metrics of various speech task models are displayed in Table 5. The MAE and RMSE are metrics for measuring prediction error which means that the lower the MAE and RMSE, the greater the predictability. With an MAE of 4.62, and RMSE of 5.85, the recall task achieved the best prediction accuracy. With an MAE of 4.89, and RMSE of 6.16, the interview task had the second-best performance.

**Table 5:** A comparison of the performance of MMSE prediction models.

Task	MAE	RMSE
Interview	4.89	6.16
Repetition	5.19	6.41
Recall	**4.62**	**5.85**

AE, mean absolute error; RMSE, root mean squared error; The performances of MMSE prediction of three speech tasks are presented. A weighted voting regressor combining random forest, ridge, and k-nearest-neighbor was used. MAE and RMSE are metrics for the measurement of prediction error and the lower these metrics, the greater the predictability.

The best results are given in bold.

## 4. Discussion

The study’s key findings were as follows: (1) AD patients exhibited speech characteristics that distinguished them from healthy controls; (2) the memory load imposed by the speech task accentuated the speech characteristics of AD; and (3) the speech task with a high memory load enhanced the performance of classification and prediction models for speech-based AD detection.

In this study, we examined the acoustic features of speech in patients with AD and healthy older adults, and investigated how these features change under different speech tasks and memory loads. We found that AD patients had distinct speech characteristics compared to healthy older adults, including a lower pitch, increased pitch variability, decreased loudness, monotonous loudness, and a slower speech rate. The present study’s findings align with those of earlier investigations, which also noted changes in the acoustic characteristics of individuals with AD (Horley et al., 2010; Martínez-Sánchez et al., 2012; Meilán et al., 2012, 2014). However, there was a disparity in regards to the frequency-related characteristics of AD. Previous studies found a higher pitch associated with AD, which is inconsistent with the current study’s results (Martínez-Sánchez et al., 2012; Meilán et al., 2014). We posit that this inconsistency is attributable to the disparate sex ratios of study participants (the proportion of female patients = 62.2% in this study; the proportion of female patients = 68% in Meilán’s study; and the proportion of female patients = 84% in Martínez-Sánchez’s study). As sex is among the most influential physiological factors affecting pitch (Puts et al., 2012), and the effects of cognitive decline and aging on speech features vary according to sex, accounting for the sex ratio of participants is critical when comparing the frequency-related features of diverse studies. Consequently, previous studies with a comparably higher female ratio than the current study yielded opposing results. Collectively, the results of the current study validate that speech’s acoustic features are associated with AD and specify how these features change, suggesting a basis for speech-based AD detection in clinical settings.

This study also demonstrated that the speech characteristics of Alzheimer’s Disease (AD) can be accentuated when a high memory load is imposed. Specifically, our findings indicate that the acoustic features of speech in AD can vary in prominence depending on the speech task, with the recall task, which involves a high memory load, eliciting the most noticeable differences. These results corroborate prior studies that have established a relationship between cognitive load and alterations in the acoustic features of speech. Previous investigations have shown that cognitive load can modify speech acoustics and that cognitively impaired individuals, including older adults, are particularly vulnerable to these effects, leading to more pronounced changes in speech characteristics (Ramig and Ringel, 1983; Kemper et al., 2010, 2011; Marzullo et al., 2010; Bailey and Dromey, 2015; MacPherson, 2019). This phenomenon can be explained by the cognitive “supply and demand” model (Seidler et al., 2010), whereby an imbalance between available cognitive resources and the cognitive demands of a task leads to greater alterations in speech motor performances and acoustic features. Temporal features, however, appear to be less affected by memory load than frequency and intensity-related features. Numerous studies have reported that a slower speech rate is the most characteristic speech trait of AD (Hoffmann et al., 2009; Martínez-Sánchez et al., 2012, 2013; Meilán et al., 2012, 2014). As a slower speech rate is one of the most recognizable acoustic features of AD, we believe it was observed to be less task-dependent. In other words, among the various speech characteristics of AD, some features are consistently distinct and not task-dependent, whereas others can be observable or unobservable depending on the context. Therefore, to induce and effectively capture the latter, we must impose a heavy memory load; consequently, cognitive struggles of AD patients can manifest as changes in the acoustic features of speech.

Last, the current investigation revealed that a speech task with a high-memory-load can improve the performance of AD classification and MMSE prediction models. We conducted a comparative analysis of the performance of AD classification and MMSE prediction models, utilizing speech data obtained from three speech tasks that varied in their memory loads. The recall task with a high memory load outperformed the other tasks in both the AD classification and MMSE prediction models that other tasks with less memory load. Few studies have compared the performance of speech tasks and evaluated their potential, and a recent study found that the recall task achieved the best classification performance using linguistic features (Clarke et al., 2021). Our study provides evidence that recall tasks employing acoustic features can effectively perform in both AD classification and MMSE prediction. It is conceivable that the acoustic features of AD, which were accentuated by the high memory load of the recall task, could increase the variability in speech characteristics between groups and facilitate their classification and prediction. Based on the obtained results, we conclude that the recall task has practical implications as a speech-based AD detection tool.

This research has a number of limitations. One limitation is the small sample size, with speech data collected from only 45 AD patients and 44 healthy controls. This limited dataset size may constrain the ability to achieve improved classification and model performance. Also, the current study conducted only one call per participant which is not free from the fluctuations in the cognitive performance of patients. Hence, building larger datasets with more participants and repeated conduction of the experiment may allow for the enhancement of classification and prediction performances and the reliability of results. Another limitation of this research is that the acoustic features were extracted using an existing conventional feature set, eGeMAPS. While eGeMAPS comprises standardized features with a robust theoretical foundation and is widely used in speech-based AD detection, it may not be the optimal feature set to describe the AD voice, as it was initially designed to identify the emotional states of a speaker. Therefore, in future studies, it is critical not only to identify the optimal speech task but also to develop an appropriate acoustic feature set that accurately characterizes the AD voice. Another limitation is that the current study provides limited explanation between the speech features and the memory load. We observed that the differences in speech features between AD patients and healthy older adults accentuated in the recall task with high memory load and it possibly led to the excellent AD classification performance and MMSE prediction performance of the recall task. Yet, a clinically valid explanation which connects the speech characteristics of AD patients and the memory load is still lacking. Hence, in the future study, the associations between speech features and the cognitive function or brain structures should be identified to provide clinical validity as an AD detection method to the speech features and the memory load.

Otherwise, this study highlights the significance of utilizing appropriate speech tasks for accurate and efficient speech-based AD detection. Our findings demonstrate that a recall task with a high memory load can effectively reveal the speech characteristics of AD, leading to improved AD classification and MMSE prediction. Given the practicality and limited availability of data and time in the real-world setting, an efficient speech-based AD detection tool must accurately detect AD with minimal resources. The use of memory load in speech tasks is shown to be advantageous in achieving effective speech-based AD detection, as it enhances the speech characteristics of AD and improves detection accuracy. Hence, rather than the combination of various speech tasks like previous studies, the compact method with high-memory-load speech task would allow the efficient and less demanding speech-based AD detection for patients and clinicians.

In addition, this study benefits from the utilization of acoustic features in speech analysis. Unlike linguistic features, which are limited to specific language properties and contexts, acoustic features serve as language-universal markers with broader applicability. Our research, based on acoustic features, allows for more generalizable findings that can be applied to various linguistic contexts.

In future research, it is important to seek neuroscientific evidence to support speech-based AD detection. While it is clear that AD patients exhibit changes in acoustic features of their speech, the neural mechanisms underlying these alterations are not yet well-understood. Thus, future investigations should explore the neural correlates of AD speech characteristics to provide clinical validity to speech-based AD detection. By elucidating the neural underpinnings of AD speech features, we can gain a better understanding of the AD and develop more accurate and effective diagnostic tools.

## Data availability statement

The raw data supporting the conclusions of this article will be made available by the authors, without undue reservation.

## Ethics statement

The studies involving human participants were reviewed and approved by the SMG-SNU Boramae Medical Center. Written informed consent for participation was not required for this study in accordance with the national legislation and the institutional requirements.

## Author contributions

J-YL, HK, and MB conceived and designed the study. MB, M-GS, and HH acquired the data. MB analyzed the data and wrote the manuscript. J-YL, KK, and MB interpreted the data. All authors have read and agreed to the published version of the manuscript.

## Funding

This research was funded by the Ministry of Education through the National Research Foundation of Korea (NRF), grant number (NRF-2021R1A2C2095809).

## Conflict of interest

The authors declare that the research was conducted in the absence of any commercial or financial relationships that could be construed as a potential conflict of interest.

## Publisher’s note

All claims expressed in this article are solely those of the authors and do not necessarily represent those of their affiliated organizations, or those of the publisher, the editors and the reviewers. Any product that may be evaluated in this article, or claim that may be made by its manufacturer, is not guaranteed or endorsed by the publisher.
